# Reduced exploration capacity despite brain volume increase in warm-acclimated common minnow

**DOI:** 10.1242/jeb.223453

**Published:** 2020-06-04

**Authors:** Libor Závorka, Barbara Koeck, Tiffany A. Armstrong, Mustafa Soğanci, Amélie Crespel, Shaun S. Killen

**Affiliations:** 1 Institute of Biodiversity, Animal Health & Comparative Medicine, Graham Kerr Building, College of Medical, Veterinary & Life Sciences, University of Glasgow, Glasgow G12 8QQ, UK; 2 WasserCluster Lunz–Inter-University Centre for Aquatic Ecosystem Research, A-3293 Lunz am See, Austria

**Keywords:** Boldness, Neural plasticity, Metabolism, Phenotypic syndrome, Global warming

## Abstract

While evidence suggests that warming may impact cognition of ectotherms, the underlying mechanisms remain poorly understood. A possible but rarely considered mechanism is that the metabolic response of ectotherms to warming is associated with changes in brain morphology and function. Here, we compared aerobic metabolism, brain volume, boldness and accuracy of maze solving of common minnows (*Phoxinus phoxinus*) acclimated for 8 months to either their current optimal natural (14°C) or warm (20°C) water temperature. Metabolic rates indicated increased energy expenditure in warm-acclimated fish, but also at least partial thermal compensation as warm-acclimated fish maintained high aerobic scope. Warm-acclimated fish had larger brains than cool-acclimated fish. The volume of the dorsal medulla relative to the overall brain size was larger in warm- than in cool-acclimated fish, but the proportion of other brain regions did not differ between the temperature treatments. Warm-acclimated fish did not differ in boldness but made more errors than cool-acclimated fish in exploring the maze across four trials. Inter-individual differences in the number of exploration errors were repeatable across the four trials of the maze test. Our findings suggest that in warm environments, maintaining a high aerobic scope, which is important for the performance of physically demanding tasks, can come at the cost of changes in brain morphology and impairment of the capacity to explore novel environments. This trade-off could have strong fitness implications for wild ectotherms.

## Abstract (precis)

**Highlighted Article:** In fish, thermal metabolic compensation in response to warming under predicted climate change scenarios may alter brain morphology and cognition, potentially causing fitness impairment.

## INTRODUCTION

In ectotherms, acute exposure to warmer temperatures causes an increase in the minimum oxygen uptake rate required to sustain life (i.e. a proxy for standard metabolic rate, SMR), which may not be matched by a similar increase in maximum oxygen uptake rate (i.e. maximum metabolic rate, MMR), leading to a decrease of aerobic scope (AS), i.e. a decrease in the capacity to deliver oxygen to support aerobic physiological processes above maintenance including activity, digestion, growth and reproduction (Sandblom et al., 2014; Nati et al., 2016). When chronically exposed to warmer temperatures, ectotherms are capable of at least partial metabolic thermal compensation, i.e. reducing their SMR and maintaining AS (Stillman, 2003; Sandblom et al., 2014; 2016; Seebacher et al., 2015). However, the cost of acclimation to chronic warming is yet to be understood. Evidence suggests that warm acclimation, for example, reduces the performance of mitochondria in fish brain cells more profoundly than in other organs, such as the heart (Chung et al., 2017). Thermal acclimation may therefore specifically affect the growth and function of the brain and cognitive skills of ectotherms, which is important as an increasing number of wild ectotherms including teleost fishes are being exposed to temperatures above their physiological optimum (Comte and Olden, 2017).

The latest evidence indicates that environmental warming can affect cognition of fish and other ectotherms. Increased temperatures have, for example, been shown to impair learning in lizards (Dayananda and Webb, 2017) and accelerate the onset of age-related learning deficits in fish (Valenzano et al., 2006), but also to improve learning capacity in juvenile sharks (Pouca et al., 2019). One of the most ecologically important cognitive skills in fish is spatial orientation, which allows individuals to efficiently explore heterogeneous environments composed of different biologically important areas (Hughes and Blight, 1999; Braithwaite, 2005). Previous work suggests that brain size and the size of brain regions is, in general, positively correlated with cognitive skills of fishes (Kotrschal et al., 2013; Pike et al., 2018). In particular, the telencephalic-tectal-cerebellar neural complex has been identified as a brain region responsible for spatial orientation (Broglio et al., 2003; Odling-Smee and Braithwaite, 2003a). However, there is currently a scarcity of studies testing the effect of warm acclimation on brain volume and its regions and the consequences of such changes on the capacity of fish to explore novel environments.

Warming has also been shown to increase the expression of energetically demanding behaviours in ectotherms including boldness, activity and aggressiveness (Biro et al., 2009; Briffa et al., 2013). This positive relationship is likely to be limited at extremely high temperatures, which can cause a range of adverse physiological effects, including altered metabolic function and diminished scope for aerobic and neuromuscular performance, which would lead to a reduced expression of all behavioural traits (Killen et al., 2013). Boldness and associated behavioural traits (e.g. activity and aggressiveness) are often directly related to life-history trade-offs between resource acquisition and survival (David and Dall, 2016). Previous work also suggests that specific cognitive skills related to spatial orientation and exploration of novel environments can be positively associated with boldness (Carazo et al., 2014; Kareklas et al., 2017).

In the present study, we compared aerobic metabolism (i.e. SMR, MMR, AS), total brain volume and that of its regions, behavioural traits (i.e. boldness, activity and aggressiveness) and capacity to explore a maze to find a reward of common minnows (*Phoxinus phoxinus*) acclimated for 8 months to either: (1) a cool water temperature simulating their current optimal natural temperature, i.e. 14°C (Cui and Wootton, 1989); or (2) a warm water temperature simulating a severe climate warming scenario (IPCC, 2013), i.e. temperature increment of 6°C to 20°C. We predicted that: (i) warm-acclimated fish would have a higher SMR and MMR but a similar AS to cool-acclimated fish; (ii) warm-acclimated fish would be more bold, active and aggressive; and (iii) warm acclimation would affect the size of the brain and its regions, but owing to a scarcity of previous studies on that topic we have no predictions regarding the direction of the effects; however, we expected that (iv) exploration capacity would increase with increasing brain and telencephalon size, and with increasing boldness of individuals.

## MATERIALS AND METHODS

### Fish sampling and housing

In November 2017, young of the year common minnows, *Phoxinus phoxinus* (Linnaeus 1758) (*N*=80, fork length, FL ∼15 mm) were collected from the River Kelvin (Glasgow, UK) by dip netting, and transferred to the nearby aquarium facilities of the University of Glasgow. All experimental fish were collected at a single sampling spot in an artificial side channel along the River Kelvin. Small minnows accumulate in this channel as water and fish pass over a small weir from the main river and small fish are then are unable to return. Breeding-size fish have never been recorded in this location as they can quickly return to the main stream via the opposite end of the channel. As such, the experimental fish were probably from a single cohort from the river population consisting of representatives from numerous families. Sex of individuals was not determined, but they were juveniles and did not reach sexual maturity until the end of the study. The common minnow occurs in lakes and rivers ranging from mountainous headwaters to small rivers at intermediate altitude with average summer water temperature lower than 25°C (Raffard et al., 2019). Evidence from bioenergetic models suggests that the optimal growth temperature of common minnow is 14°C (Cui and Wootton, 1989); this also corresponds to the average summer temperature in the River Kelvin. Climate on the western coast of Scotland is typified by a relatively low seasonal and daily fluctuation, with mean air temperature in winter ∼4°C and in summer ∼14°C. The maximum day-average water temperature in the River Kelvin recorded in 2016 was 21°C (Marine Scotland; http://marine.gov.scot/node/15086).

Collected minnows were randomly split into four groups and housed in four identical rectangular rearing tanks (32 l, 40×40×30 cm, 20 individuals per tank), which contained artificial plants and gravel substrate, and were supplied with UV-treated, re-circulating freshwater. Photoperiod followed cycles of 12 h of light and 12 h of dark. From the beginning of the laboratory period, water temperature was 14°C in rearing tanks 1 and 3 and 20°C in rearing tanks 2 and 4. Individuals were fed daily until apparent satiation (i.e. fish were given as much feed as they would consume within a 10 min period) with a mix of frozen bloodworms and aquarium flake food (warm-acclimated fish required ∼50% more feed than cool-acclimated fish). After 2 months of initial acclimation period (because of the small capture size), on 31 January and 1 February 2018, all individuals were anaesthetized in a benzocaine solution, and measured for FL to the nearest millimetre and body mass (*M*_b_) to the nearest 0.01 g (mean±s.d. FL 57±4 mm, *M*_b_ 1.98±0.48 g). Fish were then tagged with Visible Implant Elastomer (VIE; Northwest Marine Technology Inc.) with a unique colour combination for individual identification (colour combination of four colours: pink, green, red and white) before being returned to their rearing tanks, where they were left undisturbed until the start of metabolic assays. Fish were monitored daily so that individuals with visibly poor health or condition could be removed. We had to remove 1 and 4 individuals in the 14°C treatment and 2 and 7 individuals in the 18°C treatment. The procedures described in this paper comply with animal care guidelines approved within the UK and were conducted under Home Office Project 60/4461.

*M*_b_, FL and condition factor of individuals were measured on three different occasions (i.e. at tagging on 31 January to 1 February 2018, at metabolic assays on 16 to 27 July 2018, and after the behavioural assays at the end of the experiment on 2 to 11 August 2018). *M*_b_ and FL were measured to the nearest 0.1 g and millimetre, respectively. Condition factor *K* was calculated as:(1)
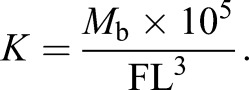
Differences in *M*_b_, FL and *K* were tested by a linear model with temperature treatment as an independent variable. To avoid type I errors associated with multiple comparisons, *P*-values of models were adjusted for false discovery rate (Benjamini and Hochberg, 1995). We found no significant difference in *M*_b_ of individuals between the temperature treatments measured at tagging (*F*_1,61_=1.496, *P*=0.678), at the time of the metabolic assays (*F*_1,61_=0.0672, *P*=0.796), or at the end of the experiment (*F*_1,64_=0.087, *P*=0.796; Fig. S1A). We found that warm-acclimated fish had significantly larger FL at tagging (*F*_1,61_=8.4332, *P*=0.0153), but there was no significant difference in FL between the temperature treatments at the time of the metabolic assays (*F*_1,61_=2.0366, *P*=0.1586), or at the end of the experiment (*F*_1,64_=3.7176, *P*=0.0875; Fig. S1B). Warm-acclimated individuals had significantly lower condition factor than cool-acclimated individuals throughout the study (at tagging: *F*_1,61_=15.852, *P*=0.0002; at metabolic assays: *F*_1,61_=16.634, *P*=0.0001; at the end of the experiment: *F*_1,64_=22.755, *P*<0.0001; Fig. S1C).

### Metabolic rates and AS

SMR and MMR of individuals (*N*=66) was estimated from the rate of oxygen uptake (*Ṁ*_O_2__) using intermittent flow respirometry (Clark et al., 2013). The metabolic assays were conducted between 16 and 27 July 2018, after 8 months of exposure to the temperature treatments. Assays took place in 16 glass chambers (0.0893 l) submerged into a water bath (92 l, 80×40×29 cm), which allowed 16 fish to be measured simultaneously. A peristaltic pump (Masterflex L/S 100 RPM, Cole-Parmer, Vernon Hills, IL, USA) was used to create a continuous mixing circuit (0.1 l min^−1^) in the water bath. Bacterial oxygen consumption was kept at a minimum by using a UV filter sterilizer and it was evaluated daily before and after the fish were place to the respirometric chambers. Measured *Ṁ*_O_2__ was then adjusted by assuming a linear increase in bacterial metabolism over the time of the assay. A thermostatic reservoir connected to the water bath by a thermoregulator (TMP-REG system, Loligo Systems, Tjele, Denmark) maintained the temperature in the water bath at the acclimation temperature of each treatment (i.e. either 14 or 20°C). Flush pumps, connected to a timer, flushed oxygenated water through the chambers for 2 min with 8 min intervals between flushes, during which the oxygen uptake of individuals was measured. Oxygen concentration within each glass chamber was recorded every 2 s by a fibre optic sensor using a 4-channel FireSting O_2_ system (PyroScience GmbH, Aachen, Germany), which was calibrated in accordance with the supplier's manual. The software package respR (Harianto et al., 2019) was used to analyse *Ṁ*_O_2__ (mg O_2_ h^−1^) data obtained from the FireSting O_2_ software. Oxygen uptake data were corrected for the length and radius of tubing, the volume of the respirometry chambers and the volume of the fish. Prior to measurement, individuals were fasted for 48 h and then randomly collected from their rearing tanks by dip nets and transferred to a temporary holding tank (circular bucket 11 l). MMR was measured as the oxygen uptake immediately after an exhaustive exercise protocol where fish were individually chased for 2 min in a circular tank containing aerated freshwater with temperature corresponding to their treatment (Clark et al., 2013; Killen, 2014). SMR was measured as the lowest 10th percentile of oxygen uptake measurements that were recorded over the time the fish were in the respirometers (∼18 h overnight), excluding the first 5 h of measurements during which the oxygen consumption is often elevated (Killen, 2014). AS was calculated as the difference between SMR and MMR. After respirometry, all individuals were measured for FL to the nearest millimetre and *M*_b_ to the nearest 0.01 g. The change in metabolic rates with an increase in temperature was described by the *Q*_10_ indicator, which is derived from the van't Hoff equation (Sandblom et al., 2014; Seebacher et al., 2015):(2)
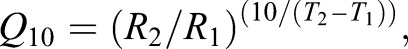
where *R*_1_ is the mean mass-specific SMR (i.e. mg O_2_ h^−1^ g^−1^ *M*_b_) of cool-acclimated individuals, *R*_2_ is the mean mass-specific SMR of warm-acclimated individuals, *T*_1_ is 14°C (i.e. cool acclimation treatment) and *T*_2_ is 20°C (i.e. warm acclimation treatment). A *Q*_10_ for SMR <2 is indicative of thermal compensation in warm-acclimated individuals (Sandblom et al., 2014; Seebacher et al., 2015).

### Behavioural measurements

**Fig. 1.**
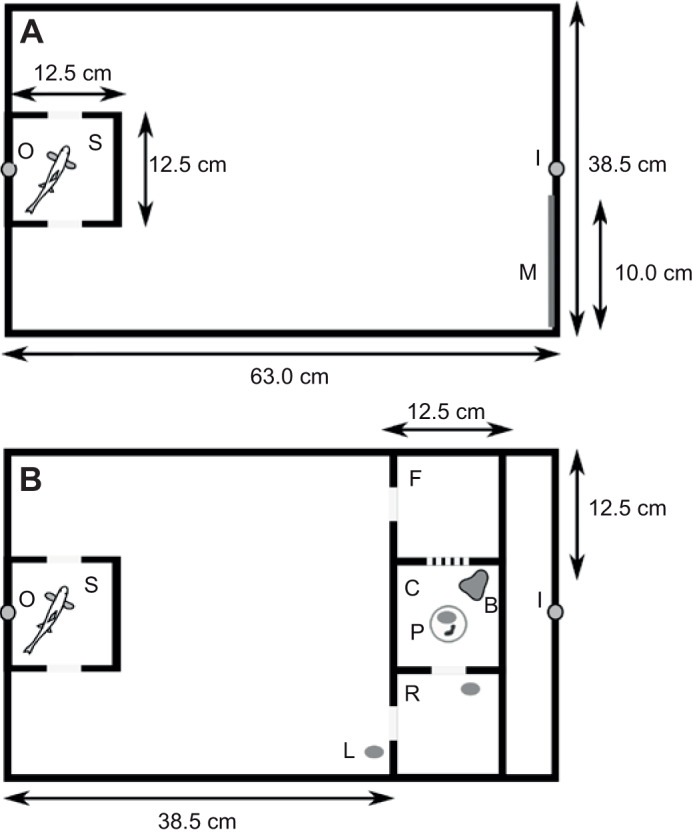
**Experimental set-up.** Diagram of the trial tank for behavioural scoring (A) and the maze test (B), viewed from above. S, starting box; M, mirror; F, maze chamber with blocked entrance; R, maze chamber with open entrance; C, central chamber; L, landmarks (gravel pieces); P, Petri dish with bloodworms; B, bait bag with bloodworms; I, water inlet; O, water outlet.

Behaviour and spatial orientation of individuals (*N*=66) were tested from 2 to 11 August 2018. Every scoring day started at 08:30 h and finished at 19:00 h. A single batch with a maximum of eight individuals was tested on each scoring day using eight flow-through (∼2 l min^−1^) rectangular transparent trial tanks (13 l, 63×38.5×20 cm, water level 5.5 cm; Fig. 1A) filled with aerated freshwater maintained at the acclimation temperature for the treatments. Trial tanks were positioned beneath four cameras (HD Webcam C525, Logitech) and lit by a dispersed dim LED light. Fish were left undisturbed in the starting box of trial tanks for 30 min after introduction into the tank, whereupon the doors on the sides of the starting box were gently lifted and the time until fish emerged from the starting box into the (empty) trial tank was measured for 50 min to evaluate the boldness of individuals (Näslund et al., 2015). Individuals that did not emerge (17 individuals out of 66) during this period were assigned the maximum time score, i.e. 50 min (3000 s). The starting box was then removed from the trial tank and the distance moved by the fish in the trial tank (i.e. open field test) was recorded over 10 min (after 10 min acclimation following removal of the starting box) to evaluate individual activity (Závorka et al., 2015). After the open field test, a mirror (10×10 cm) was slid into a corner of the trial tank and the amount of time spent in front of the mirror (i.e. within ∼2 body lengths) was measured over 10 min (after 10 min acclimation) to evaluate responses of individuals to conspecifics (Adriaenssens and Johnsson, 2013). Here, we considered the response to conspecifics as aggressiveness; however, depending on the motivation of an individual to approach the conspecific in the mirror, it could also be considered sociality (non-aggressive social interaction). The distance moved and the time spent in front of the mirror were derived from the video records by placing a grid net of 10×6 equal-sized rectangles (rectangle size 6.3×6.4 cm) over the record of the trial tank on a computer screen. Distance moved was measured, following the example of previous studies (Závorka et al., 2015; Näslund et al., 2017), as the number of crossings between rectangles, where each crossing represents a complete passage by an individual over the borderline into an adjacent rectangle. The three behavioural traits were summarized using principal component analysis, resulting in a significant first principal component (eigenvalue=1.68), which explained 56.01% of the total variance in the three behavioural tests and which was negatively correlated with time to emerge from the starting box (*R*=−0.64, loading=−0.49), and positively correlated with activity (*R*=0.78, loading=0.60) and aggressiveness/sociality towards the mirror (*R*=0.81, loading=0.63). The second and third principal component yield by PCA had eigenvalues <1 and therefore were not further considered (Fig. S2). The first principal component is hereafter named the ‘boldness score’ and interpreted as a continuum of behavioural types ranging from inactive, unaggressive and shy individuals (i.e. low scores) to active, aggressive and bold individuals (i.e. high scores) (David and Dall, 2016).

Exploration capacity was evaluated in a maze test, which challenges the spatial orientation of tested individuals (Johnsson and Sundström, 2007; Adriaenssens and Johnsson, 2011). The maze test was conducted after the mirror image test, when the starting box was returned to the trial tank and individuals were gently guided into it by a dip net without being netted or lifted out of the water. The maze, which was inserted into the trial tank while focal fish were in the starting box, was composed of three chambers, of which two were connected by an opening, allowing the individual to enter the central chamber and consume bloodworms, which served as a reward (Fig. 1B). The other chamber was connected to the central chamber by a blocked entrance that allowed the scent of the bloodworms to disperse through but prevented fish from entering the central chamber. The scent of bloodworms was amplified by placing a bait bag with bloodworms in the central chamber of the maze. This protocol eliminates the potential effect of conditioning (positive or negative) related to the reward scent on the performance of individuals in the maze, because odour had the same intensity in the two side chambers of the maze. To minimize the effect of lateralization (Bisazza and Brown, 2011), mazes in half of the trial tanks had the correct entrance on the left side and in the other half of the trial tanks had the correct entrance on the right side. The correct path to the centre of the maze was marked by gravel pieces as landmarks, which allowed individuals to use allocentric information to navigate in the maze along with the egocentric information (Rodriguez et al., 1994). Fish are known to use both types of spatial information. However, as our focal fish originated from a dynamically changing riverine environment, which limits the usefulness of allocentric landmarks for navigation (Odling-Smee and Braithwaite, 2003b), it can be assumed that they predominantly relied on egocentric information for navigation. Performance of fish in the maze test was scored in four consecutive trials. Fish were guided back to the starting box at the end of each trial and left there to rest for 20 min before the following trial started. During this period, the maze was cleaned of leftover bloodworms and prepared for the following trial. The maze trial started by gently lifting the doors and removing the cover of the starting box. Fish performance in the maze was then observed for 80 min (4800 s) in each trial. Two variables were recorded in each trial: (i) speed of maze exploration, i.e. time until entry into the central chamber of the maze after emergence from the starting box; and (ii) accuracy of maze exploration, i.e. the number of errors during exploration until entry into the central chamber of the maze with reward. Time until entry into the central chamber was assigned the highest score (i.e. 4800 s) for individuals that did not reach the central chamber until the end of the trial (49 out of 248 trials evaluated; 15 trials were not evaluated because of a technical failure). The number of exploration errors was measured as the number of compartment swaps before an individual first entered the centre of the maze minus 3, which corresponds to the number of compartments to reach the central chamber without detour. To control for feeding motivation of focal fish, their voracity was tested after the last maze trial by recording whether they consumed a set amount of bloodworms (∼0.1 g) placed in the starting box over a period of 30 min.

### Brain measurements

Immediately following the completion of the last maze trial, fish were killed with an overdose of benzocaine and their final *M*_b_ and FL were measured to the nearest 0.01 g and millimetre, respectively. Heads of fish were removed and fixed in 4% buffered (pH 6.9) paraformaldehyde solution. Brains were then dissected out as described in Gonda et al. (2009) and stored in 4% buffered paraformaldehyde solution until further procedure. Dissection was performed by opening the skull along the anteroposterior axis and removal of muscle tissue and bones around the brain until the brain could be lifted up from the skull. Note that we were not able to dissect brain from 11 individuals of the quality required for further analysis and therefore we removed them from the dataset. Brains were imaged with a Canon EOS 1300D DSLR camera with EF-S18-55 III lens (Canon) and 13 and 31 mm extension tubes designed for Canon DSLRs (Xit Inc.). For each dissected brain sample (*N*=55), an image was taken using the dorsal, left lateral and ventral views. Each brain was measured to calculate total volume and the volumes of the dorsal medulla, cerebellum, optic tectum, telencephalon and hypothalamus. Measurements were completed using ImageJ 1.48 (Schneider et al., 2012) and used to calculate volume with the formulas outlined by Pollen et al. (2007).

### Statistical analysis

All analyses were conducted in R v.3.5.3 (http://www.R-project.org/). The structure of final models is summarized in Table 1. Allometry was controlled for by adding *M*_b_ as a co-variable in all models. For brain regions, allometry was controlled for by adding the total brain volume minus the volume of the region of interest as a co-variable (Pike et al., 2018; Table 1). Generalized linear mixed effect models for data with Poisson and Gaussian distribution were respectively used to analyse the number of errors and the time of entry into the central chamber of the maze (Bates et al., 2015). In the models for boldness score and spatial orientation (i.e. capacity to explore a novel environment), allometric residuals of aerobic scope (resAS) and whole brain (resWhole brain) and telencephalon (resTelencephalon) were used to avoid collinearity of these covariables with *M*_b_. Repeatability of an individual's performance in the maze test was tested as the random slope of an individual's ID across the four maze test trials (Stoffel et al., 2017). Repeatability of between-individual differences was tested on a subset of 33 individuals that successfully solved the maze test in all four trials. We used 1000 bootstraps to calculate the 95% confidence interval (CI) of repeatability estimates. Effect of acclimation temperature on feeding motivation was tested using the chi-square test.

**Table 1.**
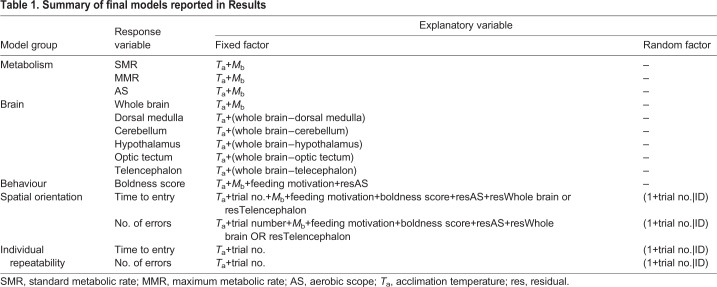
**Summary of final models** **reported in Results**

Note that the use of only two rearing tanks in each temperature treatment could reduce the independence of temperature treatments on random differences between the holding tanks. Therefore, all models initially included rearing tank as a random intercept to control for pseudo-replication effect, despite the fact that this variable had a lower number of levels (four) than the minimum recommended for random effects (>5–6; Bolker et al., 2009). However, this variable was removed from all final models as it did not significantly improve the model fit, i.e. ΔAIC >2 (Burnham and Anderson, 2004). This suggests that we can conclude with caution that the effect of rearing tanks on our results was probably negligible. Significance of the models was evaluated using ANOVA tables using Type II sums of squares. Model fit was diagnosed by control of distribution of model residuals and association of fitted and residual values. Differences among trials of the maze test were analysed using Tukey's HSD *post hoc* test.

## RESULTS

**Fig. 2.**
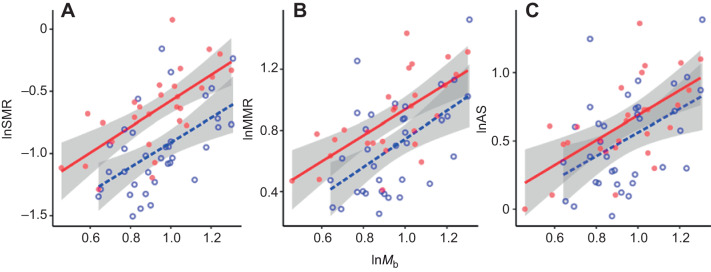
**Correlation of body mass** **(*M*_b_) with metabolic rate and aerobic scope (AS) for fish in the two temperature treatments.** (A) Standard metabolic rate (SMR; mg O_2_ h^−1^), (B) maximum metabolic rate (MMR; mg O_2_ h^−1^) and (C) AS (mg O_2_ h^−1^) against *M*_b_ (g) for fish acclimated to warm (20°C, red filled circles and solid curves; *N*=28) and optimal temperature (14°C, blue open circles and dashed curves; *N*=35). Circles represent individual data points. Shaded areas represent s.e.m.

Warm-acclimated fish had a higher SMR (*F*_1,60_=23.70, *P*<0.001; Fig. 2A) and MMR (*F*_1,60_=10.68, *P*=0.002; Fig. 2B) than cool-acclimated fish, but AS did not differ between the temperature treatments (*F*_1,60_=2.77, *P*=0.101; Fig. 2C). SMR (*F*_1,60_=34.28, *P*<0.001), MMR (*F*_1,60_=32.82, *P*<0.001) and AS (*F*_1,60_=22.23, *P*<0.001) were positively correlated with *M*_b_ in both temperature treatments. *Q*_10_ for mean mass-specific SMR between cool and warm-acclimated individuals was 1.69. Together, the absence of a difference in AS between fish from cold and warm temperature treatments and a *Q*_10_ of <2 indicate partial metabolic thermal compensation of the warm-acclimated fish.

**Fig. 3.**
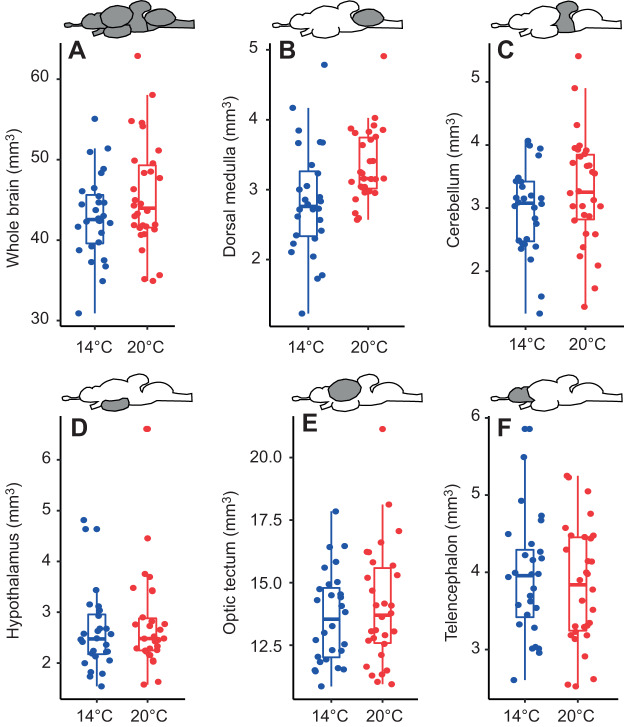
**Brain volume of fish in the two temperature treatments.** Boxplots illustrating differences in whole-brain volume (A) and that of its functional regions (B, dorsal medulla; C, cerebellum; D, hypothalamus; E, optic tectum; F, telencephalon) for fish acclimated to warm (20°C, red circles; *N*=30) and optimal temperature (14°C, blue circles; *N*=28). Displayed data are raw measurements not corrected for *M*_b_ and whole-brain size of individuals. Central lines represent the median, box limits are 25th and 75th percentiles and whiskers cover the 95th percentiles. Filled circles represent individual data points.

We found that whole-brain volume was larger in warm-acclimated than in cool-acclimated individuals (*F*_1,55_=7.21, *P*=0.010; Fig. 3A) and positively correlated with *M*_b_ in both treatments (*F*_1,55_=57.04, *P*<0.001). Volume of the dorsal medulla was larger in warm-acclimated than in cool-acclimated fish (*F*_1,55_=8.22, *P*=0.006; Fig. 3B). The volume of the cerebellum (*F*_1,55_=2.43, *P*=0.125; Fig. 3C), hypothalamus (*F*_1,55_=0.18, *P*=0.671; Fig. 3D), optic tectum (*F*_1,55_=0.05, *P*=0.816; Fig. 3E) and telencephalon (*F*_1,55_=2.33, *P*=0.132; Fig. 3F) was not affected by acclimation temperature. The volume of all brain regions was positively correlated with whole-brain volume (i.e. *P*<0.05), with the exception of cerebellum volume (*F*_1,55_=0.07, *P*=0.79).

**Fig. 4.**
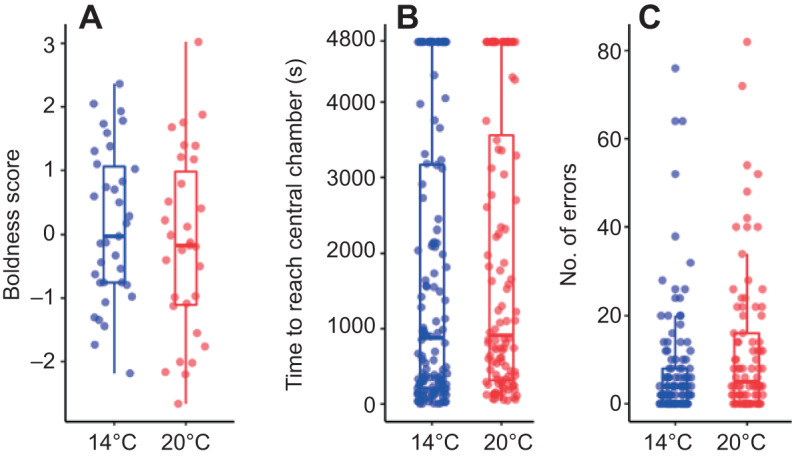
**Behaviour and spatial orientation of fish in the two temperature treatments.** Boxplots illustrating differences in behaviour (boldness; A) and maze performance (time to reach the central chamber and number of errors; B,C) of fish acclimated to warm (20°C, red filled circles; *N*=31) and optimal temperature (14°C, blue filled circles; *N*=35). Central lines represent the median, box limits are 25th and 75th percentiles and whiskers cover the 95th percentiles. Filled circles represent individual data points.

Boldness score was positively correlated with *M*_b_ (*F*_1,55_=10.29, *P*=0.002) and tended to be lower in warm- than in cool-acclimated individuals, albeit this difference was not significant (*F*_1,55_=3.73, *P*=0.059; Fig. 4A). There was no effect of feeding motivation (*F*_1,55_=0.98, *P*=0.337) and residual AS (*F*_1,55_=2.14, *P*=0.148) on boldness score.

Individuals with higher boldness score (χ^2^=19.41, *P*<0.001) and with larger *M*_b_ (χ^2^=7.29, *P*=0.007) entered the central chamber of the maze faster than individuals with low boldness score and low *M*_b_. Time to entry into the central chamber of the maze was not significantly related to acclimation temperature (χ^2^=0.01, *P*=0.918; Fig. 4B), trial number (χ^2^=3.14, *P*=0.371), feeding motivation (χ^2^=0.120, *P*=0.913), residual AS (χ^2^=0.00, *P*=0.99), residual whole-brain volume (χ^2^=3.39, *P*=0.066) or residual telencephalon volume (χ^2^=0.032, *P*=0.855). Inter-individual differences in time until entry into the central chamber across the four maze test trials were not repeatable [*R*=0.08, 95% CI (0.00; 0.29)].

The number of errors during the maze exploration was higher in fish acclimated to warm temperature (χ^2^=5.46, *P*=0.019; Fig. 4C) and it significantly differed between the four trials of the maze test (χ^2^=129.53, *P*>0.001). However, the number of errors did not differ between the first and the last trial of the maze test, either in warm- (*post hoc P*=0.831) or cool-acclimated (*post hoc P*=0.99) individuals. This indicates that individuals did not learn to explore the maze more efficiently over time. The number of errors was not significantly related to *M*_b_ (χ^2^=0.24, *P*=0.627), boldness score (χ^2^=0.33, *P*=0.565), feeding motivation (χ^2^=2.94, *P*=0.086), residual AS (χ^2^=0.04, *P*=0.838), residual whole-brain volume (χ^2^=0.42, *P*=0.516) or residual telencephalon volume (χ^2^=0.92, *P*=0.338). Individual differences in the number of errors across the four maze test trials were moderately repeatable [*R*=0.28, 95% CI (0.22; 0.77)].

## DISCUSSION

Our study shows that warming can reduce the capacity of fish to explore novel environments, possibly as a result of the deterioration of short-term working spatial memory (Hughes and Blight, 1999) rather than differences in spatial learning skills (Braithwaite, 2005). In fact, inter-individual differences in the number of exploration errors during the maze test were repeatable, but we also observed no difference in the number of errors between the first and the last round of the maze test, indicating that in both treatments fish did not learn to locate the reward from previous trials. Working memory is known to drive algorithmic searching which facilitates the search for resources in a rapidly changing environment (Hughes and Blight, 1999). However, warm-acclimated fish showed a higher tendency for stereotypic movements, moving repeatedly in and out of a chamber before exploring the rest of the maze, indicating that warming may negatively affect the algorithmic searching patterns that fish use to locate prey and shelter in rapidly changing landscapes such as the riverine environment (Odling-Smee and Braithwaite, 2003b).

In our study, boldness score was not associated with the number of exploration errors, which does not support the finding of previous studies that boldness is positively associated with spatial orientation and the capacity to efficiently explore novel environments (Carazo et al., 2014; Kareklas et al., 2017). The lack of association between boldness and number of exploration errors also corroborates that individual differences in stress response to isolation and novel environment in the maze test were unlikely to cause the differences in searching patterns between the temperature treatments (Conrad et al., 2003; Závorka et al., 2019). Our results showed that bolder and larger individuals entered the central chamber of the maze earlier and thus support the suggestion that bolder individuals sample the environment faster but not more efficiently than shy ones, which could provide them with more information about the distribution of resources at the cost of a higher predation risk (Sih and Del Giudice, 2012).

In contrast to previous studies showing that brain size is positively correlated to cognitive ability (Kotrschal et al., 2013; Marhounová et al., 2019), we found that warm-acclimated fish made more errors during the maze exploration despite having a larger brain, including a disproportionately larger dorsal medulla than cool-acclimated fish. This suggests that the increment of brain volume, associated with thermal compensation of warm-acclimated fish, did not lead to the expansion of functional brain tissue (e.g. number of neurons; Olkowicz et al., 2016; Marhounová et al., 2019). The fish brain, although representing only about 2% of *M*_b_, is an energetically demanding organ, responsible for 5–15% of basal whole-animal oxygen uptake (Nilsson, 1996; Moran et al., 2015). As the availability of oxygen for glucose metabolism during and after peaks of neuronal activity is the predominant limiting factor for function and development of the fish brain (Soengas and Aldegunde, 2002; Shulman et al., 2004), the metabolic response of fish to environmental warming (i.e. thermal acclimation) may have affected brain function. We found that warm-acclimated fish had higher SMR, but similar AS to cool-acclimated fish because of partial thermal acclimation. Previous work indicates that the contribution of the brain to overall oxygen uptake relative to other organs could be reduced under warm acclimation (Chung et al., 2017). In our study, oxygen uptake of the brain in warm-acclimated individuals could have been further reduced by their lower energetic status (i.e. lower condition factor despite ∼50% higher food intake than cool-acclimated fish), because low energetic status has been shown to substantially reduce glucose oxidation in fish brain (Soengas and Aldegunde, 2002). The larger dorsal medulla in warm-acclimated individuals could also contribute to differences between cool- and warm-acclimated individuals in oxygen uptake, because it is a brain region responsible for the regulation of respiratory functions in fish (Woldring and Dirken, 1951).

We have demonstrated the effects of warming on the plastic phenotypic response within a single cohort of individuals at a single life stage. Further investigations are therefore needed to fully appreciate the implications of potential climate-driven cognitive changes in wild animals. The relative ecological importance of cognitive changes should, for instance, be compared with other changes in behaviour (Cooper et al., 2018) and physiology (Killen, 2014), and their associations (Raffard et al., 2019) induced by warming, e.g. in the context of social interactions (Jolles et al., 2019). Long-term transgenerational studies are needed to deepen our understanding of the chronic effects of warming on animal cognition and the adaptive capacity of wild fish and other ectotherms. Temporal and spatial fluctuation of temperature in their natural habitat may provide ectotherms with a thermal refuge in a warming climate (Horký et al., 2019; Morash et al., 2018). Yet, it is not known how optimal thermal habitat selection could be affected by cognitive impairment of the individual. An important avenue for future ecological research would be to investigate the synergistic effects of temperature-induced cognitive impairment and other factors known to affect the behaviour of individuals, such as parasitic load (Wengström et al., 2016), sex (Carazo et al., 2014) and life history (Goulet et al., 2017).

In conclusion, this study provides novel insight into the mechanistic association between simple cognition skills and whole-organism physiology under a climate warming scenario. Overall, our results confirm that thermal metabolic compensation in response to warm temperatures can help individuals to maintain a high capacity for aerobic metabolism (i.e. high AS) to perform energetically demanding behaviours (e.g. boldness, aggressiveness and activity), which are also shown to be directly linked to ecologically relevant behaviours in the wild such as foraging, territory defence and predator avoidance (Závorka et al., 2015; Metcalfe et al., 2016). However, our findings also indicate that thermal compensation of ectotherms in response to warming can alter brain morphology and function, potentially causing cognitive impairment, probably affecting fitness (Boogert et al., 2018) and ecological interactions (Edmunds et al., 2016).
